# Time trends in treated incidence, sociodemographic risk factors and comorbidities: a Finnish nationwide study on anxiety disorders

**DOI:** 10.1186/s12888-022-03743-3

**Published:** 2022-02-22

**Authors:** Prakash Khanal, Tiia Ståhlberg, Terhi Luntamo, David Gyllenberg, Kim Kronström, Auli Suominen, Andre Sourander

**Affiliations:** 1 Research Centre for Child Psychiatry, University of Turku, Turku, Finland; 2 INVEST Research Flagship Center, University of Turku, Turku, Finland; 3 Department of Adolescent Psychiatry, Turku University Hospital, Turku, Finland; 4 Department of Child Psychiatry, Turku University Hospital, Turku, Finland; 5 National Institute of Health and Welfare, Helsinki, Finland; 6 Department of Adolescent Psychiatry, Helsinki University and Helsinki University Central Hospital, Helsinki, Finland

**Keywords:** Anxiety disorders, Treated incidence, Risk factors, Comorbidities

## Abstract

**Background:**

There has been a lack of research about the time trends and socio-demographic risk factors for children and adolescents who receive treatment for anxiety disorders. This study aimed to fill these gaps in our knowledge by examining a nationwide sample of Finnish children and adolescents diagnosed in specialized healthcare settings.

**Methods:**

This study comprised national register data of all singleton children born in Finland from 1992–2006 who were diagnosed with anxiety disorders from 1998–2012. The changes in time trends in incidence were studied by dividing the study sample into three cohorts by birth years: 1992–1996, 1997–2001 and 2002–2006, who were followed up until the age of 20, 15 and 10 years, respectively. The 22,388 individuals with anxiety disorders were age and gender matched with 76,139 controls from the general population. Logistic regression was used to examine the socio-demographic risk factors and anxiety disorders in the entire sample. Comorbid disorders were examined in the oldest birth cohort (1992–1996 born).

**Results:**

Comparing the 1992–1996 and 2002–2006 cohorts showed that the cumulative incidence of treated anxiety disorders at the age of 10 increased from 0.3 to 1.2% among females and 0.46 to 1.9% among males. Subjects had higher likelihood for being diagnosed with an anxiety disorder if their mothers had low maternal socio-economic status class at birth (OR 1.53, 95% CI 1.45–1.61) compared to higher SES class, and marital status was single at the time of birth (OR 2.02, 95% CI 1.87–2.17) compared to married or in a relationship. They had lower risk of anxiety disorders diagnosis if born in rural (OR 0.82, 95% CI 0.79–0.86) or semi-urban areas (OR 0.79, 95% CI 0.76–0.82) when compared to urban residence. There was a wide range of psychiatric comorbidities, and unipolar depression was the most common (31.2%).

**Conclusion:**

Anxiety disorders diagnosed by specialized Finnish services increased from 1998–2012 in both genders. This could indicate a real increase in overall anxiety disorders or an increase in treatment seeking. The findings on maternal socioeconomic status and single parenting improve the recognition of the environmental risk factors for anxiety disorders among children and adolescents.

## Background

Anxiety disorders are very common in childhood and adolescence and global estimates suggest they affect 6.5% of individuals under 19 years of age [1]. The prevalence varies for different anxiety disorders and so does the usual age at onset. Child onset disorders usually include separation anxiety, specific phobias and selective mutism, whereas social phobia, agoraphobia, panic disorder and generalized anxiety disorder are more likely to start in adolescence [2]. Anxiety disorders cause significant functional impairment at home, at school and with friends [3]. They can manifest as somatic symptoms [3, 4] and increase the risk of several other psychiatric disorders, which lead to further deterioration in the prognosis [5, 6]. When they are left untreated, anxiety disorders tend to become chronic [7] and may even lead to disabilities [8]. Even with their widespread prevalence, the treatment rates for anxiety disorders are low [9]. These issues highlight how important it is to recognise the risk factors for anxiety disorders, so that children and adolescents can be diagnosed and treated as early as possible.

Previous time trend studies for anxiety disorders have showed a phenomenon of increased health care visits [10–14] especially by girls [10, 11, 14]. However, these studies had limitations with regard to sample sizes, definition of diagnoses and study periods. In addition, a number only compared two cohorts and this introduced the risk that any changes could have been down to natural fluctuations, rather than specific factors. Time trend studies provide information for health care planning and identify which factors need to be further investigated when changes are observed. These can include different cultural and lifestyle aspects and sociodemographic structures.

The aetiology of anxiety disorders comprises both genetic and environmental risk factors [2, 15]. The existing studies on environmental risk factors, such as sociodemographic risk factors, are mostly cross-sectional or small cohort studies, and only few studies have followed a prospective design. There have been a few larger register-based studies that have focused on socio-demographic factors, such as parental unemployment [16], welfare benefits [17], low maternal socio-economic status (SES) [18], family breakdowns and single parents [16–18]. These factors have all been associated with a higher risk for anxiety disorders. In addition, two Danish registry studies found that the risk for diagnosed anxiety disorders was higher among those living in urban areas [16, 19]. However, all the previous studies have had either very narrow inclusion criteria for anxiety disorders [18, 19] or a wide selection of diagnoses, which included obsessive compulsive disorder and post-traumatic stress disorder [16, 17]. These are not considered anxiety disorders in the Diagnostic and Statistical Manual of Mental Disorders, 5th Edition, as their aetiology is different [20]. Moreover, all these studies had relatively small sample sizes compared to the present study.

Finland has well-established national registers, which contain valuable psychiatric epidemiological data [21–23]. The aim of our study was to provide a comprehensive description of the anxiety disorders observed among a large sample of children and adolescents being cared for by specialized Finnish mental health services. We used three birth cohorts to study the treated incidence, cumulative incidence and time trends and examined the socio-demographic factors and psychiatric comorbidities associated with diagnosed anxiety disorders.

## Materials and methods

This nationwide register-based case-control study is a part of Finnish Prenatal Study of Anxiety Disorders. It included information on all singleton children born in Finland between 1 January 1992 and 31 December 2006 and followed up until 31 December 2016. The cases included individuals who were diagnosed with an anxiety disorder between 1 January 1998 and 31 December 2012. The study used a number of Finnish nationwide registers that provided comprehensive information on various datasets.

### National registers

The Finnish registers used in this study were the Care Register for Health Care recognized also as Finnish Hospital Discharge Register, the Central Population Register and the Finnish Medical Birth Register. Detailed descriptions of these registers have previously been published [22, 23] and are briefly explained here.

The Care Register includes information on all visits to specialized health services in Finland. From 1969 to 1998, this database contained information from all inpatient wards in somatic and psychiatric hospitals, local health care centres, private hospitals, prison hospitals and military wards. Since 1998, it has also included outpatient diagnoses. This study used the Care Register to identify cases diagnosed with anxiety disorders by specialized mental health services and to collect any comorbid diagnoses that were recorded during the study period. During our study period the diagnoses were coded using the International Classification of Diseases, Tenth Revision (ICD-10). The Care Register is a continuation of the Discharge Register, and its inclusiveness and accuracy have been assessed to be satisfactory to very good [24].

The Population Register provides information on all Finnish citizens and permanent residents and it is maintained by the Population Register Centre and local register offices. It includes information such as the individual’s name, address, data of birth and death, family members and any immigration or emigration status. In this study the Population Register was used to collect information on two risk factors for the cases and matched controls, namely the place of birth and whether someone lived in an urban or non-urban area.

The Birth Register was established in 1987 and it contains standardized data on the prenatal and perinatal periods of infants who are born alive and stillborn. In this study the Register was used to collect information on the mother’s socio-economic and marital status at the time of her child’s birth. The database has previously been described [25].

The linkage of information between these registers was possible using the personal identity code issued for each Finnish resident at birth or when they move to the country. The approval for the utilization and linkage of the register data was provided by the Data Protection Ombudsman and National Institute for Health and Welfare, Finland, and the ethical approval was obtained from the Ethics Committee of the Hospital District of Southwest Finland. This study is solely based on register data hence obtaining informed consent was not required by the ethics committee. This study was done in accordance to the research guidelines and regulations of National Institute for Health and Welfare, Finland.

### Study participants

#### Cases and controls

This study comprises of a nested case-control sample. It used national register data of all singleton children born in Finland in 1992–2006. There were 22,388 cases identified from the Care Register, who were diagnosed with anxiety disorders from 1998 to 2012. The sample was further divided into three birth cohorts, 1992–1996 (13,806 cases), 1997–2001 (6453 cases) and 2002–2006 born individuals (2129 cases). These individuals were followed up until the age of 20, 15 and 10 years, respectively.

Anxiety disorders were defined using the ICD-10 classifications of: agoraphobia (F40.0, F40.00, F40.01), social phobia (F40.1, F93.2), specific phobias (F40.2, F40.8, F40.9, F93.1), panic disorder (F41.0, F41.00, F41.01, F41.08, F41.09), generalized anxiety disorder (F41.1, F93.80), separation anxiety (F93.0), selective mutism (F94.0) and other, nonspecific anxiety disorders (F41.2, F41.3, F41.8, F41.9, F93.3, F93.89, F93.9). This study includes cases that were diagnosed with any anxiety disorders at least once when they were at least six years old. We excluded obsessive compulsive disorder and stress-related disorders and cases diagnosed with moderate or severe intellectual disability (ICD-10: F72 and F73).

The cases were matched with controls from the Population Register by their date of birth (± 30 days), and gender. The controls were still living in Finland when their matched cases were first diagnosed with an anxiety disorder. The controls had not been diagnosed with any anxiety disorder or moderate to severe intellectual disability (ICD-10 F72, F73) during the study period. A total of 76,139 controls were included in the analyses.

### Demographic variables

Maternal SES and marital status at birth were obtained from the Birth Register. Maternal SES was divided into the five maternal occupation categories used by Gissler et al. [26]. These were upper white collar (including experts and managers), lower white collar (including clerical workers), blue collar (including manual workers), others (including entrepreneurs, students, people who were unemployed and stay-at-home parents) and missing data. This classification is also the national Finnish classification for occupations and socio-economic groups. The region of birth and urbanicity were obtained from the Population Register and defined as Southern, Eastern, Northern and Western Finland. These areas were further broken down by urban, semi-urban or rural, based on the definitions from Statistics Finland. Finland is one of the mostly sparsely populated countries in Europe. According to Statistics Finland, the average population density is 18 inhabitants per square kilometres, varying from 0.2 in some areas in the north to almost 3000 in the capital city. In semi-urban areas the inhabitant rate varies from 6 to 62 [27].

### Comorbidities

The comorbid psychiatric diagnoses were retrieved from the Care Register for the oldest cohort born in 1992–1996 in order to have longer follow-up period. Diagnoses at 6–16 years of age were included to make the sample uniform. The comorbidities were defined using the ICD-10 classification of mental disorders (F10-F99), excluding anxiety disorders (Table 1).

**Table 1:** ICD-10 diagnostic codes for comorbid psychiatric diagnosis

Substance abuse disorders (F10-F19), schizophrenia spectrum disorders (F20-F25, F28 and F29), bipolar disorders (F30 and F31), unipolar depressive disorders F32-F34, F38 and F39), obsessive-compulsive disorders (F42), stress-related and dissociative disorders (F43 and F44), somatoform disorders (F45), eating disorders (F50), non-organic sleep disorders (F51, excluding F51.3 and F51.4), personality and habit-impulse disorders (F60-F63), intellectual disability (F70-F79, excluding F72 and F73), learning and coordination disorders (F80-F83), autism spectrum disorders (F84), attention deficit hyperactivity disorder (F90), conduct and oppositional disorders (F91 and F92), attachment disorders of childhood (F94.1 and F94.2) and tic disorders (F95).	

### Statistical analyses

We studied the time trends of the treated incidence and cumulative incidence of diagnosed anxiety disorders, by birth year: 1992–1996, 1997–2001 and 2002–2006. First, we analysed the changes in incidence rates among diagnosed anxiety disorder cases. The population at risk were children living in Finland at the end of 2012 and were obtained from Population Register. The yearly incidence of diagnosed anxiety disorders per 100 people at risk was calculated by gender. Second, the yearly cumulative incidence was calculated with odds ratios (OR) and 95% confidence intervals (95% CI). The cumulative incidence was one minus the estimated survival function, which was calculated using the Kaplan-Meier estimate. Third, both the yearly incidence and cumulative incidence were calculated separately for the three birth cohorts, at 20, 15 and 10 years of age, respectively. The statistical difference between the treated incidence rates for males and females was calculated using the log-rank test. In addition, we examined if there were any cases that received anxiety disorder diagnoses at the age before six and was not followed up after age six (n = 88).

We used nested case-control design to examine the possible association between sociodemographic factors and anxiety disorders in the entire sample. Conditional logistic regression was used to examine the unadjusted associations in the total sample and by gender. The ORs and 95% CIs were calculated for each variable and the outcome was an anxiety disorder diagnosis. Then adjustments were made for significant covariates, p-value <0.05 was considered to indicate statistical significance. We examined the randomness of the missing values among the risk factors and used multiple imputation method as missing values were non-random. Twenty imputed datasets were produced and logistic regression model was fitted with each dataset. These results were pooled to obtain the final results. The frequencies for the anxiety disorder subgroups in the total population and comorbid diagnoses in the oldest cohort, born in 1992–1996, were calculated. Chi-square test was done to see the differences in the prevalence of comorbid diagnoses between genders. The statistical analyses including multiple imputation were performed using SAS statistical software, version 9.4. (SAS Institute Inc., Cary, NC, USA).

## Results

### Characteristics of the study population

The characteristics of the study population is presented in Table 2. The total sample comprised of 98,527 children and adolescents. Of these, 22,388 were cases diagnosed with anxiety disorders, of which 56.4% were female. The mean age at diagnosis was 11.3 years (standard deviation (SD) 2.9, age range 6–20 years). The mean age was 11.6 years (SD 3.1) for females and 10.9 years (SD 2.7) for males. For the three birth cohorts, 1992–1996, 1997–2001 and 2002–2006, the mean ages for the females and males were 15.5 and 14.4 (SD 2.5, 3.3, range 6–20 years), 11.6 and 10.5 (SD 2.5, 2.2, range 6–15 years) and 7.7 and 7.8 (SD 1.3, 1.2, range 6–10 years), respectively.

**Table 2:** Gender distribution and mean age of anxiety disorder diagnosis in the whole sample and in the three birth cohorts

Cohorts	Total n of cases *Mean age at first diagnosis (SD)*	Female n (%) *Mean age (SD)*	Male n (%) *Mean age (SD)*
Entire sample 1992–2006 (6–20 years)	22,388 *11.3 (2.9)*	12,628 (56.4%) *11.6 (3.1)*	9760 (43.6%) *10.9 (2.7)*
Birth cohort 1992–1996 (6–20 years)	13,806 *15.1 (2.9)*	8793 (63.7%) *15.5 (2.5)*	5013 (36.3%) *14.4 (3.3)*
Birth cohort 1997–2001 (6–15 years)	6453 *11.0 (2.4)*	3043 (47.2%) *11.6 (2.5)*	3410 (52.8%) *10.5 (2.2)*
Birth cohort 2002–2006 (6–10 years)	2129 *7.8 (1.3)*	792 (37.2%) *7.7 (1.3)*	1337 (62.8%) *7.8 (1.2)*

### Yearly incidence of diagnosed anxiety disorders by age and gender

**Fig. 1:**
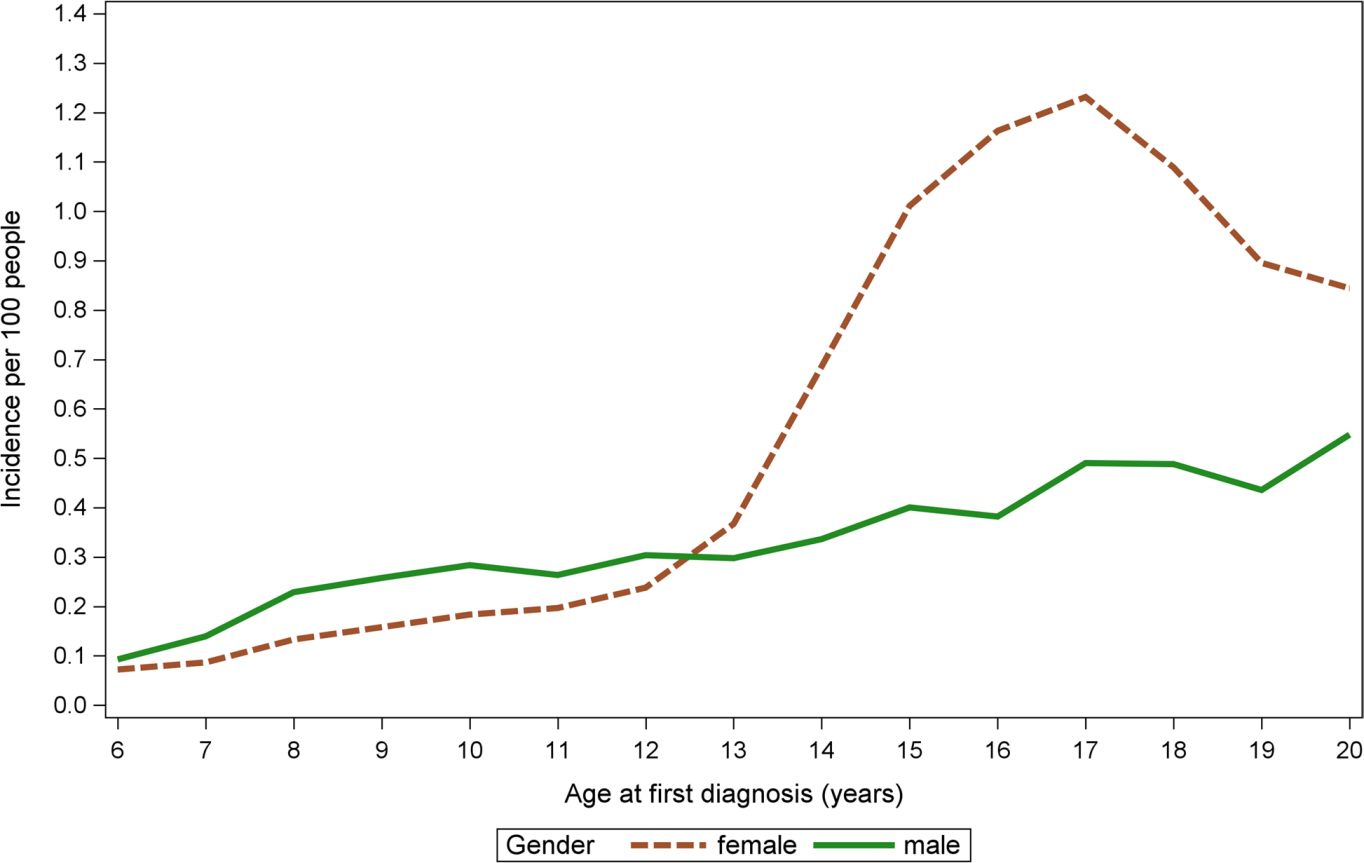
Yearly incidence of diagnosed anxiety disorders by age and gender per 100 people

The age-specific changes in diagnosed anxiety disorders were examined by analyzing the yearly incidence by both age and gender (Fig. 1). The incidence showed a notable increase among females from 12.5–17 years of age, whereas the increase in incidence was smaller and practically stable in males from 6–20 years of age. Males had more anxiety disorders at 6–12 years of age than females (p < 0.001) and for females it was from 13–20 years of age than males (p < 0.001).

### Cumulative incidence of diagnosed anxiety disorders by birth cohort and gender

**Fig. 2:**
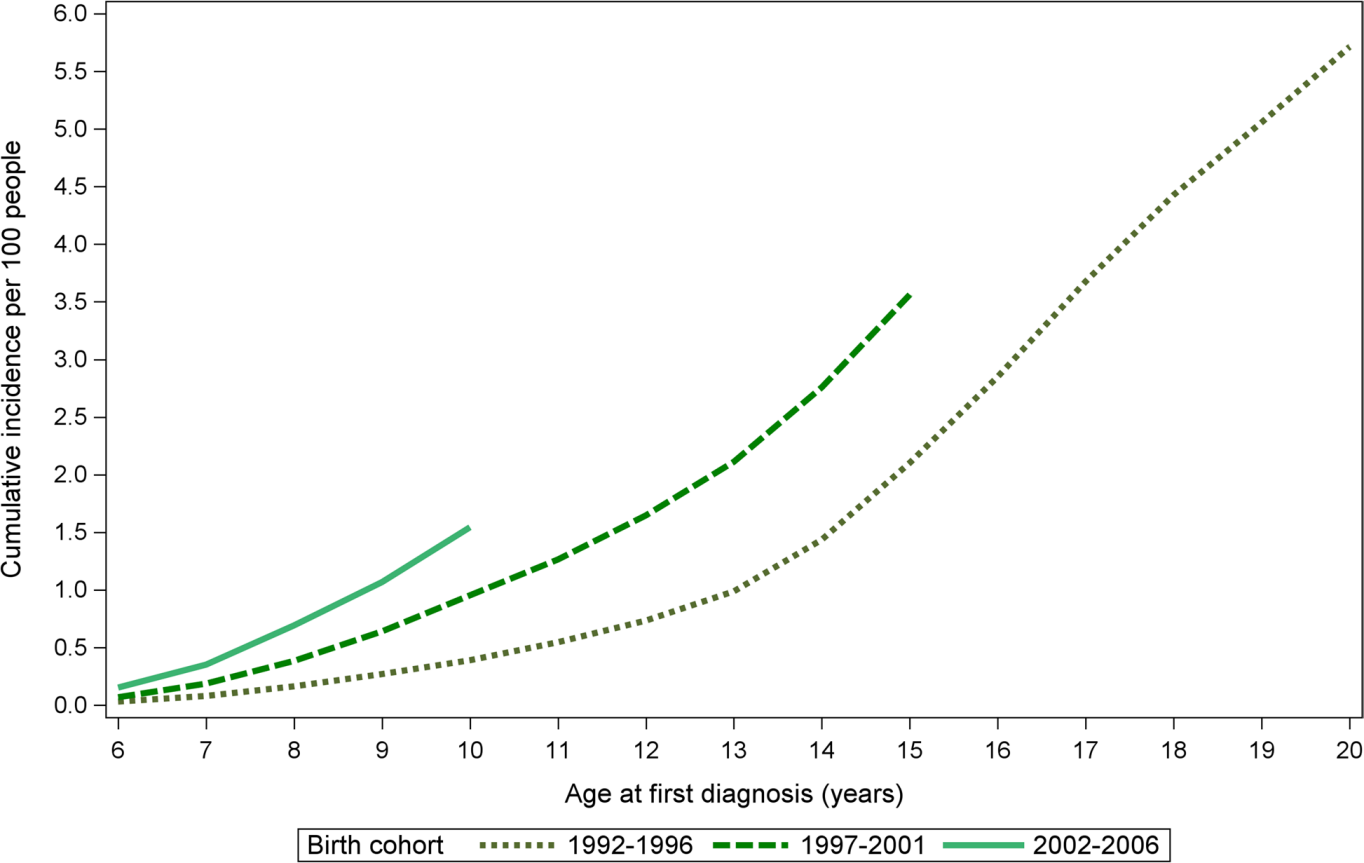
Cumulative incidence of subjects diagnosed by birth cohort per 100 people

**Fig. 3:**
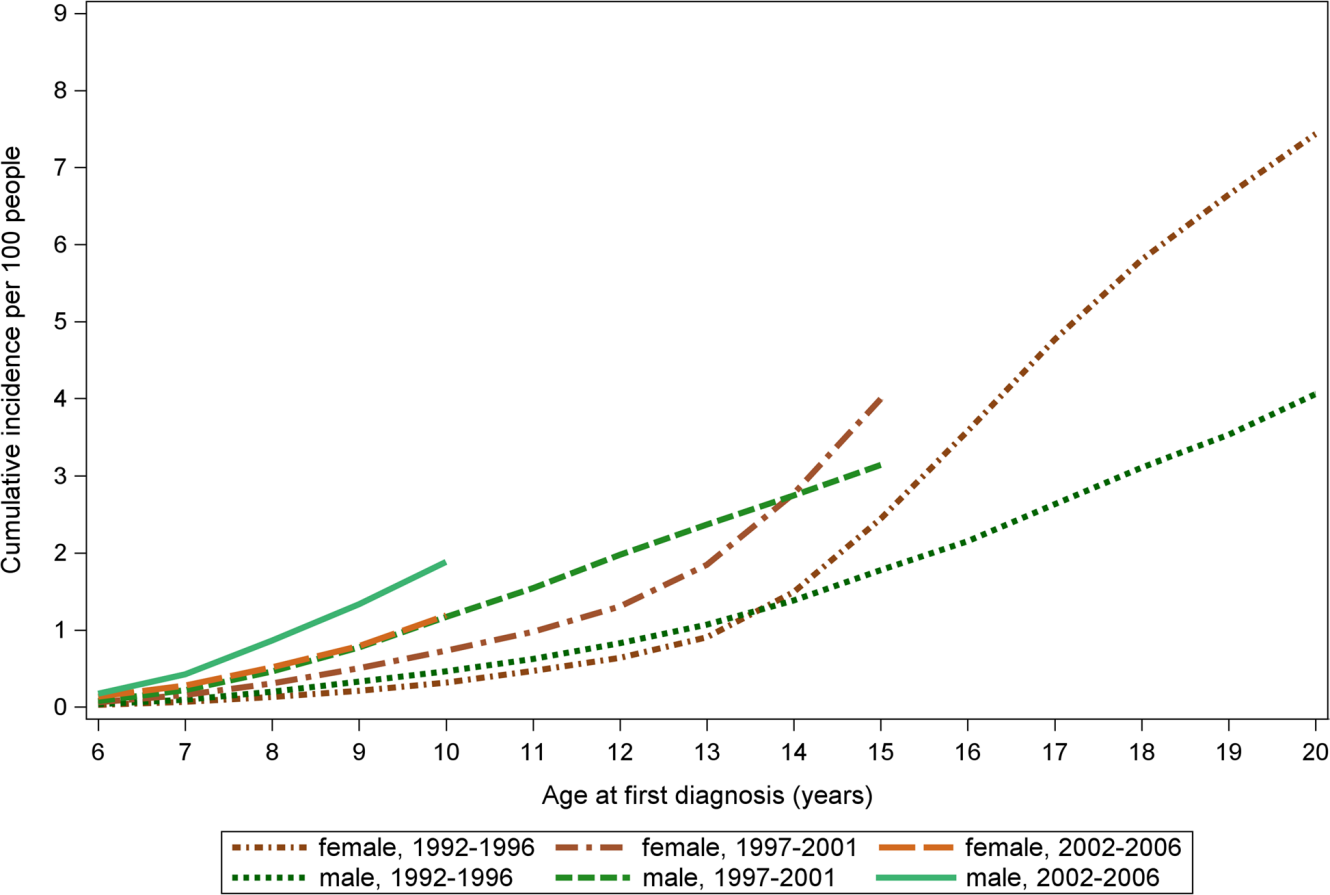
Cumulative incidence of diagnosed anxiety disorders by gender and birth cohort per 100 people

Figures 2 and 3 show the cumulative incidences of diagnosed anxiety disorders per 100 live births by birth cohort and gender. The first birth cohort was followed up until the age of 20 years and the other cohorts were followed until 15 and 10 years of age, respectively. Individuals born in 1992–1996, and diagnosed with anxiety disorders by the age of 20, had cumulative incidences of 5.7% (95% CI 5.60–5.82) by the age of 20 and 0.4% (95% CI 0.36–0.41) by the age of 10. The incidence had increased in the 1997–2001 and 2002–2006 groups by the age of 10, with cumulative incidences of 0.9% (95% CI 0.91–0.99) and 1.5% (95% CI 1.46–1.62), respectively.

Similar trends were noticed in both genders. The cumulative incidence at the age of 10 increased when the first and last, 1992–1996 and 2002–2006, cohorts were compared. They increased from 0.3% (95% CI 0.28–0.34) to 1.18% (95% CI 1.09–1.29) among females and 0.46%, (95% CI 0.43–0.49) to 1.9% (95% CI 1.76–2.00) among males.

### Demographic factors and the risk of anxiety disorders

Table 3 shows the sociodemographic characteristics of the study sample. Of the 98,527 individuals, 5348 were missing data on maternal SES, 431 on urbanicity and 8151 on marital status. When the upper white-collar SES class was used as the reference category, children born to the mothers of blue collar (OR 1.53, 95% CI 1.45–1.61) and lower white-collar (OR 1.18, 95% CI 1.12–1.24) SES classes were more likely to get diagnosed with anxiety disorders. These results were obtained after adjusting for the other sociodemographic covariates, gender and time of birth; p < 0.0001. Another significant finding observed in the adjusted analysis was that children born to single mothers had higher likelihood of anxiety disorder diagnoses (OR 2.02, 95% CI 1.87–2.17) p < 0.0001, when compared to the reference category of children born to mothers who were married or in a relationship. The likelihood of being diagnosed with an anxiety disorder was lower in Western Finland, (OR 0.68, 95% CI 0.66–0.71), Northern Finland (OR 0.59, 95% CI 0.56–0.62) and Eastern Finland (OR 0.67, 95% CI 0.63–0.71); (p < 0.0001 for each) when Southern Finland was used as the reference category. The odds of being diagnosed with an anxiety disorder was also lower in rural, (OR 0.82, 95% CI 0.79–0.86) and semi-urban, (OR 0.79, 95% CI 0.76–0.82) (both p < 0.0001) when urban residence was used as the reference category.

**Table 3:** Demographic factors and risk of anxiety disorders

		Cases		Controls		Unadjusted			Adjusted^a^		
		N	%	N	%	OR	95% CI	***p***- value	OR	95% CI	***p***- Value
**Maternal SES Class**	Upper white-collar workers	2707	12.1	11,660	16.1	Ref			Ref		
	Lower white-collar workers	8972	42.8	34,040	47.1	**1.13**	**1.07–1.18**	**<0.0001**	**1.18**	**1.12–1.24**	**<0.0001**
	Blue collar workers	4722	22.6	14,009	19.4	**1.44**	**1.37–1.52**	**<0.0001**	**1.53**	**1.45–1.61**	**<0.0001**
	Other	4541	21.7	12,528	17.3	**1.55**	**1.47–1.64**	**<0.0001**	**1.65**	**1.56–1.74**	**<0.0001**
**Region of birth**	Southern Finland	11,331	50.6	30,192	39.7	Ref			Ref		
	Western Finland	6743	30.1	27,006	35.5	**0.66**	**0.64–0.69**	**<0.0001**	**0.68**	**0.66–0.71**	**<0.0001**
	Northern Finland	2322	10.4	10,788	14.2	**0.57**	**0.55–0.60**	**<0.0001**	**0.59**	**0.56–0.62**	**<0.0001**
	Eastern Finland	1992	8.9	8153	10.7	**0.65**	**0.61–0.68**	**<0.0001**	**0.67**	**0.63–0.71**	**<0.0001**
**Urbanicity**	Urban	15,161	68.0	46,199	60.9	Ref			Ref		
	Semi-urban	3249	14.6	13,175	17.4	**0.75**	**0.72–0.78**	**<0.0001**	**0.79**	**0.76–0.82**	**<0.0001**
	Rural	3881	17.4	16,431	21.7	**0.72**	**0.69–0.75**	**<0.0001**	**0.82**	**0.79–0.86**	**<0.0001**
**Maternal marital status**	Married/ in a relationship	18,681	93.8	68,457	97.1	Ref			Ref		
	Single	1224	6.1	2014	2.9	**2.28**	**2.11–2.45**	**<0.0001**	**2.02**	**1.87–2.17**	**<0.0001**

^a^ = The multivariate model included maternal SES class, region of birth, urbanicity, maternal marital status, gender and birth year

*Ref* Reference category, *OR* Odds ratio, *CI* Confidence interval

### Gender distribution of anxiety disorder subgroups among total cases

Most of the diagnoses were described as unspecified anxiety disorders, with separate data for females (76.6%) and males (74.1%), Other commonly diagnosed anxiety disorders were specific phobias (female 10.0%, male 13.6%), panic disorders (female 9.8%, male 5.1%), generalized anxiety disorders (female 9.1%, male 10.2%), social phobias (female 9.1%, male 8.1%) and agoraphobia (female 1.6%, male 1.2%).

### Gender differences in comorbid diagnosis in the 1992–1996 birth cohort

**Fig. 4:**
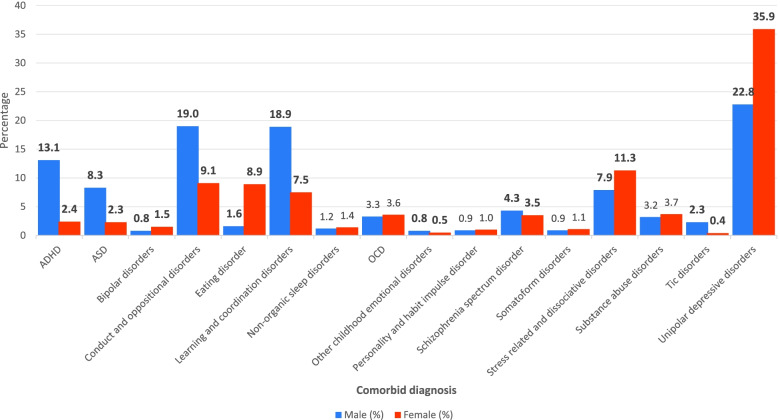
Gender differences in comorbid diagnosis in the 1992–1996 birth cohort (age 6–16 years)

We restricted the distribution and comorbidity analysis to the oldest subgroup, which were born between 1992 and 1996 and this showed that 13,806 individuals had been diagnosed with anxiety disorders (Fig. 4). The most common comorbidities among females were unipolar depressive disorders (35.9%), stress related and dissociative disorders (11.3%), conduct and oppositional disorders (9.1%), eating disorders (8.9%) and learning and coordination disorders (7.5%). Among males they were unipolar depressive disorders (22.8%), conduct and oppositional disorders (18.9%), learning and coordination disorders (18.9%) and ADHD (13.1%). Significant gender differences were observed in several comorbidities, ADHD (female 2.4%, male 13.1%), ASD (female 2.3%, male 8.1%), tic disorders (female 0.4%, male 2.3%), learning and coordination disorders (female 7.5%, male 18.9%), and eating disorders (female 8.9%, male 1.6%); p < 0.0001.

## Discussion

This nationwide birth cohort study of 22,388 children and adolescents, and matched controls, provides comprehensive data on the incidence, comorbidities and socio-demographic risk factors associated with diagnoses of anxiety disorders.

There were three key findings. First, the treated incidence of anxiety disorders increased with age, with rapid increases in females from 12.5 years, but more stable increases in males. Our data confirmed previous research that males had more anxiety disorders diagnosed in childhood, but the incidence was higher in females from adolescence onwards [28, 29]. Previous studies have discussed whether girls were more vulnerable to internalizing disorders in adolescence because of psychological, environmental, genetic, or biological factors, such as puberty, hormones and immune system regulation [29–31]. Girls appear to perceive more stress, especially from school environments and self-imposed demands [32, 33]. The timing of puberty [34] and body image concerns [35] might predispose girls to anxiety symptoms. Genders show different emotional responses to stress [36] and in how they identify and react to symptoms [37, 38], how openly they talk about their feelings [39] and how they seek help [38, 40]. This may be why girls are more likely to be treated for anxiety disorders. Another possibility is that the assessment methods tend to favour the symptoms experienced by girls [30], or that early school-based interventions for externalizing behaviour have improved more than those for internalizing symptoms, which are more common among girls [31], and hence girls could be directed more likely into specialized services. In our sample the girls’ incidence rate decreased after the age of 17. This could be due to the early occurrence of anxiety disorders, special vulnerability of girls in puberty or unspecific anxiety being part of the human development during teenage, especially among girls [2, 29, 30]. Additionally, the decrease in the incidence rates at older ages could be explained by the Finnish health care system; the treatment of anxiety disorders in adults is more concentrated in the primary health care.

We found that the cumulative treated incidence rates had increased with time among both genders, in line with previous studies [11, 13, 14]. Previous time trend studies have suggested that this increase is possibly due to increase in health care visits especially by girls [10–14]. The increased resources in mental health services in Finland have helped expanding need-based psychosocial support, treatment and rehabilitation, raising mental health awareness and change in attitude towards psychiatry in general population [11]. It is also possible that the incidence rates in our sample have increased due to prior diagnosis and the rates could be comparable at older ages. A study from UK found the increases in the incidence rates in primary care to be highest among those aged 10–16 years, not among 6 to 9-year-olds [14]. In Finland, emotional symptoms increased in female adolescents from 1988 to 2014 [41] and they fluctuated in eight-year-old children from 1989 to 2013, but with no significant increases [42]. However, service use increased in these community-based samples [41–43], especially among children with significant problems [43, 44].

Secondly, we assessed the associations between socio-demographic factors and anxiety disorders in a nested case-control setting. The risk factors appeared to be equal for both genders which includes low maternal socio-economic status, single mothers, urban living and being born in Southern Finland. Previous reports also found that low maternal SES increased the odds of anxiety disorders being diagnosed in children when the highest SES class was used as the reference [17, 45, 46]. In general, low SES has been linked with various somatic, cognitive and socioemotional problems in children [47, 48], as well as unfavorable parental behaviors and higher family stress [49, 50]. McEwen reported that children from low SES classes often experienced chronic stressful life events and the long-term allostatic load made them vulnerable and increased their risk for mental health problems, including anxiety [51].

Other studies have also reported that being a single mother increased the risk for treated anxiety disorders [17], but some did not find associations with child mental health [52, 53]. Single mothers tend to have lower incomes and education [54–56]. The wellbeing of single parents is also often compromised by a lack of social support [54, 56] and they might have less time to spend with their children [55]. More mental health problems and substance abuse have been reported among single than cohabiting mothers [52, 56, 57]. Single mothers have also been associated with controlling and dismissive parenting behaviors [53] and single parents with more childhood stress [49].

The finding that the birth region and degree of urbanicity was associated with anxiety disorder diagnoses among children also agrees with previous studies. A Danish three-generation study [19] showed that urban living was associated with a higher level of anxiety disorders than rural living. Previous studies on psychiatric disorders in adult populations [58–60] concluded that urban living was modestly, but consistently, associated with psychopathology. However, it concluded that further research was needed, particularly on mood and anxiety disorders. Urban living might contribute to the development of various mental disorders in different ways and two main hypotheses have been discussed. The drift and breeder hypotheses suggest that people either migrate selectively, and that ill people concentrate in certain areas, or that urbanicity is a stressor [61]. Urban areas are thought to have more stressors than rural areas, due to being more densely populated with smaller homes, more crime, more hectic lifestyles and pollution [59, 62]. However, urban residents could also benefit from better access to mental health services, less stigma and an increased willingness to seek help [63, 64]. Previous studies have associated urban living with a higher use of mental health services by Finnish children and adolescents [65]. Similarly, being born in Southern Finland was identified as a risk factor for anxiety disorders in our study and being born in Northern Finland had a mild protective effect. Children and adolescents use less psychiatric outpatient care services in Northern Finland and access to service has been suggested as a possible explanation for these regional differences [64].

Thirdly, we examined psychiatric comorbidities. Unipolar depression was the most common comorbid diagnosis, and this confirmed previous studies [3, 6, 66, 67]. Learning disorders was also a common comorbid diagnosis, as previously reported [68]. These finding underline the importance of assessing comorbidities when treating anxiety disorders. It is also important to avoid unintended over diagnosis [69], as this can have a significant impact on the severity and duration of symptoms [6].

Our study had some limitations that needs to be considered when interpreting the findings. First, the anxiety disorders subjects from six years of age onwards were identified from Finnish Hospital Discharge Register diagnosed by specialized mental health services. Individuals with less severe symptoms may not receive a diagnosis, end up in specialized services or not even seek for treatment. Additionally, parental factors contribute in the help-seeking of minors. Hence, information on these children and adolescents is not available in register. Our rate estimates should be interpreted as treated incidence rates and cannot be regarded as population-based incidence rates. Second, the register data did not enable addressing the differences in the incidence rates between the genders. Third, this study analyzed data on children, adolescents, and young adults and hence the results cannot be interpreted beyond the follow-up age. Since the socio-demographic data were recorded at the time of birth, we were unaware of any changes later in life. Finally, most of the anxiety disorder diagnoses were unspecified, therefore we could not study risk factors and comorbidities associated with anxiety disorder subgroups at this stage.

## Conclusion

We believe that this study covered the largest sample of children and adolescents treated by specialized mental health services for anxiety disorders to date. It remains unknown whether the increased treatment rates found in our study reflect a real increase in overall anxiety, merely increased treatment seeking or treatment seeking at earlier ages. Future studies are needed to improve our understanding of the specific reasons behind these increases. The demand for specialized mental health services for children and adolescents is increasing, which also indicates that the currently available primary care services might not be sufficient. The findings on low maternal SES and single parenting increasing the risk for anxiety disorders emphasizes the impact of environmental factors.

## Data Availability

The data that support the findings of this study are available from Finnish Social and Health Data Permit Authority- FINDATA, but restrictions apply to the availability of these data, which were used under license for the current study, and so are not publicly available. Data are however available from the authors upon reasonable request and with permission of FINDATA.

## Abbreviations

ADHD: Attention Deficit Hyperactivity Disorder
ASD: Autism Spectrum Disorder
CI: Confidence Interval
CRHC: Care Register for Health Care
DSM-5: Diagnostic and Statistical Manual of Mental Disorder, 5th Edition
ICD-9: International classification of Disease, Ninth revision
ICD-10: International classification of Disease, Tenth revision
OCD: Obsessive compulsive disorder
OR: Odds ratio
SD: Standard deviation
SES: Socioeconomic status
